# Doctors and Health

**Published:** 1899-11

**Authors:** 


### Doctors and Health.

The question, "Do doctors and health go together?" is answered in the affirmative by the *Toledo Bee*, which has editorially gone over the facts and statistics. It finds that countries having few physicians, like Russia, have the highest death-rate, while those best provided for, like Holland and the United States, have the lowest. Something might be asked as to medical service, and there is no doubt that in the 120,000, the quality of, so-classed physicians of this country, there are some who deserve no credit for this good result, but without these America is one of the best provided for countries, as to well-qualified physicians. Great Britain, nevertheless, with half as many to the population, is as well off as to health as we are, considering her crowded condition. There is certainly a surplus of doctors in this country, and considering that our profession is self-destructive in its tendency, that it cuts its own throat, so to speak, with its success, this fact only aggravates the evil. There are apparently enough doctors in the country to make it distressingly healthy.—*The Journal.*

